# A novel lncRNA SOX2OT promotes the malignancy of human colorectal cancer by interacting with miR-194-5p/SOX5 axis

**DOI:** 10.1038/s41419-021-03756-y

**Published:** 2021-05-15

**Authors:** Ye Feng, Ying Xu, Yongjian Gao, Yiying Chen, Xuefeng Wang, Zhi Chen

**Affiliations:** 1 Department of Gastrointestinal Colorectal and Anal Surgery, China–Japan Union Hospital of Jilin University, Jilin, 130033 China; 2 Department of Nephrology, First Hospital of Jilin University, Jilin, 130021 China

**Keywords:** Colorectal cancer, Cell signalling

## Abstract

Long noncoding RNAs (lncRNAs) show emerging roles in colorectal cancer (CRC) development and are considered to be involved in the potential mechanism of tumor malignancy. While Sox2 overlapping transcript (SOX2OT) has been implicated in the progression of multiple cancers, its role in CRC remains to be explored. In this study, in situ hybridization (ISH) and qRT-PCR were performed to establish the functional relationships between SOX2OT and CRC deranged in CRC tissue and cells. Subsequently, SOX2OT shRNAs vectors were transfected into CRC cells to performed loss-of-function assays to detect the potential role of SOX2OT on proliferation and metastasis in vitro and vivo. The results showed SOX2OT was an oncogene that was up-regulated in human CRC tissues and cell lines. SOX2OT silencing suppressed cell proliferation, migration, and invasion in CRC cells in vitro, and inhibited tumorigenesis in the mouse xenografts. Bioinformatic predictive analysis coupled with the dual-luciferase reporter, RNA immunoprecipitation (RIP), and functional rescue assay elucidated the mechanistic network of the SOX2OT-miR-194-5p-SOX5 axis in CRC. Mechanistically, SOX2OT acted as a competing endogenous RNA (ceRNA) to upregulate SOX5 by sponging miR-194-5p. Downregulated SOX2OT boosted miR-194-5p expression, thus decreased the protein level of SOX5, which suppresses tumorgenesis of CRC.

## Introduction

With the changes in diet structure and lifestyle, the incidence of colorectal cancer (CRC) increases globally and it has been gradually ranked the top three in morbidity worldwide^1,2^ Although significant advances in treatment have been achieved with the advance of new chemotherapeutical and biological agents, however, treatment of CRC remains a therapeutic challenge and metastasis is still the dominating cause of failure^3^. The overall 5-year survival rate of CRC was less than 50% with local and distant metastasis^4^. Therefore, an improved treatment strategy is urgently needed. Current therapies for CRC are mainly primarily surgical, chemotherapy, and radiotherapy. Therapeutic strategies targeting oncogenes and their associated pathways would significantly increase the specificity of treatments and improve efficacy in CRC therapy. To elucidate the molecular mechanism implicated in the tumorigenesis of CRC, and to carry out targeted therapy offers great promise as a novel therapeutic strategy.

In recent years, many studies have shown that non-coding RNA (ncRNA) are closely related to tumor oncogenesis. The abnormal expression of long non-coding RNA (lncRNA) and microRNA (miRNA) can be detected in almost all CRC tumor tissues^5,6^ LncRNA and miRNA are closely related to the occurrence and development of tumor, and play a very important role of oncogene or tumor suppressor gene in the development of tumor by regulating tumor cell proliferation, invasion, apoptosis and drug resistance^7,8^ A variety of lncRNAs are closely related to the occurrence and development of CRC; however, their function and mechanisms remain to be fully elucidated. SRY-box containing gene 2 (Sox2) is a key regulatory factor involving vertebrate cell stemness^9^. Sox2 overlapping transcript (SOX2OT) is a lncRNA located overlaps with the SOX2 gene in sequence^10^. It is highly conserved to participate in SOX2 regulation and demonstrated important roles in conserved ontogenetic processes^11^. Interestingly, its expression patterns and potential functions have also been confirmed in CRC previously^12^, however, the underlying mechanism of SOX2OT in CRC tumor appeals to more investigations.

As a class of lncRNAs, the molecular mechanism accounting for abnormal miRNA expression in tumor depend on it exert significant roles in gene transcriptional regulation by interacting with the lncRNA or the 3′untranslated regions (3′UTR) of target messenger RNAs (mRNAs) thus affecting the translation of the potential genes^13^. MiRNA dysregulation and its effects have been widely observed in CRC and various miRNAs have been considered potential candidates for CRC treatment target and prognosis biomarker^14,15^ In particular, miR-194 has been reported to negatively regulate various cancer including CRC and inhibit cell migration and invasion^16,17^ where it may act as a potential tumor suppressor. However, there is a lack of adequate knowledge on the regulatory mechanism of lncRNA/miRNA in biological behavior of the CRC. In the current research work, we discovered that SOX2OT was upregulated in CRC tissues and CRC cells, together with having a negative association with the miR-194-5p. Then we thoroughly investigated the molecular mechanism mediating the interaction between SOX2OT and miR-194-5p in CRC and expected to provide new ideas for the diagnosis and treatment of CRC.

## Materials and methods

### Tissue collection and cell culture conditions

CRC tissues and paired paracancerous colon tissues were obtained from twenty-eight patients who underwent colonal resection in the China–Japan Union Hospital of Jilin University from November 2018 to July 2019 and confirmed by pathological diagnosis. The age, gender, tumor size, pathological grade and other clinical data were collected and all patients in this study have signed informed consent and obtained the approval from the ethics committee. Fresh samples were quickly stored in liquid nitrogen tank. CRC cell lines HT-29, HCT-116, SW480, DLD1, and normal colon epithelial cell line NCM460 were provided by the cell bank of Shanghai Chinese Academy of Sciences (Shanghai, China).

These cells were grown in DMEM medium (Gibco, Carlsbad, CA, USA) coupled with 10% fetal bovine serum (FBS, Gibco) and 1% penicillin-streptomycin (HyClone, Logan, Utah, USA) at 37 °C and 5% CO_2_. The cells in the logarithmic growth phase were taken for a subsequent experiment.

### Total RNA isolation and quantitative reverse transcription-polymerase chain reaction (qRT-PCR)

Total RNA was isolated with Trizol reagents (Invitrogen, Carlsbad, CA, USA). The purity and content of total RNA were detected using a UV spectrophotometer and each paired sample was adjusted to the same concentration. The extracted RNA was reversely transcribed into cDNA using a reverse transcription kit (QIAGEN, Valencia, CA, USA). SYBR Premix Ex TaqTM kit (Takara, Kyoto, Japan) was used for Real-time quantitive PCR analyses according to their manufacturer’s instructions. A specific stem-loop RT primer was used for miR-194-5p. The primers primers were displayed as below: SOX2OT forward: 5′–GACTCTCGCCTGTGATGGA–3′, reverse: 5′–GGCAGCTCTGTACCTTTATTCC–3′; SOX5 forward: 5′–CATTGGCCCTGGACAGA–3′, reverse: 5′–CTACCGCTCGAAGTGGCAA–3′; U6 forward: 5′–CTCGCTTCGGCAGCACA–3′, reverse: 5′–AACGCTTCACGAATTTGCGT–3′; GAPDH forward: 5′–AACGTGTCAGTGGT GGACCTG–3′, reverse: 5′–AGTGGGTGTCGCTGTTGAAGT–3′. MiR-194-5p was normalized to U6. SOX2OT and SOX5 expression data were normalized to GAPDH. The relative expression of the target gene was determined by the 2^−△△Ct^ method.

### In situ hybridization (ISH)

The paraffin-embedded tissues were cut into 4 μm thick sections. After that, xylene and ethanol were used for dehydration, and sections heated at 56 °C for 5 min. The sections were sealed with 3% H_2_O_2_ for 10 min, hybridized with 20 μl pre hybridizing solution in 42 °C incubators for 3.5 h, and hybridized with 20 μl probe solution (synthesized by Sangon Biotech Co., Ltd., Shanghai, China) in 40 °C incubators for 20 h; The hybridization was continued for 2 h in dry environment, washed and sealed. Biotinylated rat anti-digoxin, streptavidin-biotin peroxidase complex ABC and DAB were added in turn according to the instructions of the ISH kit. Finally, the samples were observed under a light microscope

### Transfection

Scrambled shRNA of SOX2OT (Con-shRNA), SOX2OT shRNAs (SOX2OT-shRNA1 and SOX2OT-shRNA2), SOX2OT siRNA (si-SOX2OT), as well as pcDNA3.1-Control (pcDNA-Con) and pcDNA3.1-SOX2OT (pcDNA-SOX2OT) were purchased from OBIO TECHNOLOGY (Shanghai, China). The shRNAs specifically targeting SOX2OT and its corresponding control were then constructed for the lentivirus package. miR-194-5p-inhibitor (miR-inhibitor), miR-194-5p-mimic (miR-mimic), and their negative control (miR-NC) were provided by GenePharma (Shanghai, China). The efficiency of the lentiviruses and miR-mimic/miR-inhibitor were assessed before the experiments. Cells were utilized for transfection at a density of 5 × 10^5^ cells/well. Lipofectamine 2000 Reagents (Invitrogen Co., Carlsbad, CA, USA) and polybrene (Sigma-Aldrich, St-Louis, MO, USA) were used for cell transfection with lipofectamine or lentiviruses, respectively. After transfection, cells were maintained for 48 h prior to analysis.

### Cell counting kit-8 (CCK-8) assay

The transfected HT-29 and SW480 cells seeded in 96 well plates were digests with trypsin to cell suspension at a concentration of 2 × 10^4^ cells/well. In total, 10 μl CCK-8 solution (KeyGEN, Nanjing, China) was added into each well at 0, 24, 48, 72, or 96 h, and incubated at 37 °C for 2 h in the dark. The optical density (OD) values of each well at 450 nm wavelength were measured using an automatic microplate reader. The experiment was repeated three times.

### 5-ethynyl-2′-deoxyuridine (EDU) assay

Transfected CRC cells were seeded onto a 6 cm plate and incubated with 50 μM EDU for 5 h. After fixation in 4% paraformaldehyde for 30 min and washed with PBS twice. The EDU-positive cells were visualized with Cell-Light™ EdU Apollo®567 In Vitro Imaging Kit (RioBio, Guangdong, China) following the manufacturer’s guide. Subsequently, the EDU-positive cells were captured by confocal laser microscopy (Zeiss, Oberkochen, Germany).

### Cell cycle analysis

Transfected CRC cells were harvested with 40 μg/mL SLPI for 1 h, after washed with PBS for thrice, the cells were fixed with 70% ethanol overnight at 4 °C. After washing, the cells were then stained with propidium iodide staining buffer (550,825, 50 μg/mL; BD Pharmingen, USA) for half an hour at 25 °C. The cell cycle distribution was analyzed using a flow cytometer (BD Biosciences, USA).

### Cell invasion and migration assays

The in vitro assessment of migration and invasion was performed with a transwell assay. After 48 h of transfection, the cells were collected and digested with trypsin to form a single cell suspension with a cell density of 1 × 10^6^ cells/ml. For cell invasion assay, the upper and lower chambers of the Transwell chamber were separated by a polycarbonate microporous membrane coated with matrigel. Cell migration assay was performed using chambers without matrigel. In total, 100 μL cell suspension was added in the upper chamber and 500 μl complete medium containing 10% FBS was added in the lower chamber. Then the cells were incubated at 37 °C, 5% CO_2_ and saturated humidity for 24 h. Cells were then immobilized and stained with 0.1% crystal violet (Sigma-Aldrich). Under a brightfield microscope (Olympus CKX41, Tokyo, Japan), five fields were randomly selected to count the number of transmembrane cells.

### Xenograft tumor model

HT29 cells stably transfection with Con-shRNA and SOX2OT-shRNAs were dissociated using trypsin and 1 × 10^6^ cells were subcutaneously inoculated into the flank of the nude mice (5 weeks, male, BALB/c Nude) to establish the xenograft model. All experiments were performed in accordance with the Guide for the Care and Use of Laboratory Animals (NIH publication 80–23, revised 1996), with the approval of the experimental animal ethics committee of Jilin University. The tumor growth was monitored every 7 days. Three weeks later, the mice were sacrificed and the tumors samples were collected for further staining.

### Hematoxylin and eosin (HE) and immunohistochemistry (IHC) staining

The histopathology examination was performed with HE staining with Paraffin-embedded sections (4 μm thickness) as previous reported^18^. As for immunohistochemical analysis, paraffin-embedded tumor tissue sections were deparaffinized by xylene and hydrated with gradient ethanol. Then, the sections were treated with citrate buffer (pH = 6) for 20 min and immersed in 3% H_2_O_2_ for 10 min in a humidified chamber. After washing with PBS 5 min × 3 and blocked for 30 min with 10% goat serum, sections were incubated with with rabbit anti-ki67 antibody (1:400 Abcam, Cambridge, MA, USA) in a humidified chamber at 4 °C overnight with a two step protocol. Five randomly selected fields were captured under high-power magnification (200×) using a bright-field microscope (Olympus, Tokyo, Japan) and analysed using Image-Pro Plus v6.2 software (Media Cybernetics, Silver Spring, MD).

### Luciferase reporter assay

The predicted SOX2OT 3′UTR or SOX5 3′UTR fragment containing miR-194-5p binding site was inserted into pmirGLO (GenePharma Co.Ltd., Suzhou, China) to obtain wild-type Report gene vectors. The target sequence was mutated to obtain the mutant vector SOX2OT 3′UTR mut and SOX5 3′UTR mut. After co-transfection, the luciferase activity of HT-29 and SW480 cells was detected by a dual-luciferase activity assay kit 48 h later. The mean luciferase intensity was normalized to renilla luciferase.

### RNA immunoprecipitation (RIP) assay

RIP assay was performed using a Magna RIP™ RNA Binding Protein Immunoprecipitation Kit (Millipore, Bedford, MA, USA) as previously described to detect the binding potential of SOX2OT and miR-194-5p. HT-29 and SW480 cells were collected and lysed in complete RIPA buffer containing a protease inhibitor cocktail and RNase inhibitor which contained magnetic beads with antibodies of Ago2 or IgG (negative control). The protein was digested and the corresponding immunoprecipitated RNA was obtained for further RT-qPCR analysis.

### Protein extraction and western blotting

Total proteins were disintegrated by using RIPA lysis buffer (Beyotime Biotechnology, Shanghai, China) and qualified by a BCA detecting kit (Keygen, Nanjing, China) following the manufacture’s protocol. The extracted protein samples were subjected to 10% SDS-PAGE and transferred onto a PVDF membrane (GE Healthcare Bio-Sciences Corp., Piscataway, NJ), then incubated with primary antibodies of SOX5 (ab94396, 1:1000, Abcam, Cambridge, MA, UK), E-cadherin (SC-8426, 1:800, Santa Cruz Biotechnology, Dallas, TX. USA), N-cadherin (ab18203, 1:1000, Abcam), Vimentin (SC-6260, 1:2000, Santa Cruz Biotechnology), Fibronectin (SC-271098, 1:600, Santa Cruz Biotechnology) and GAPDH (ab8245, 1:2000, Abcam) at 4 °C overnight, respectively. The reacted proteins were visualized with horseradish peroxidase (HRP)-conjugated secondary antibodies for 2 h at room temperature and visualized by using ECL detection reagents (Amersham Biosciences, Pittsburg, PA, Sweden) for protein detection.

### Statistical analysis

Statistical analysis was performed using SPSS 17.0 software and each experiment was performed at least in triplicate. The one-way analysis of variance (ANOVA) was used to determine the significant difference of multiple groups and Student’s *t*-test was used to compare the significant difference of two groups.

Measured data following a normal distribution are presented as mean ± standard deviation (SD). *P* < 0.05 was considered statistically significant.

## Results

### SOX2OT is upregulated in CRC tissue and cell lines

**Fig. 1:**
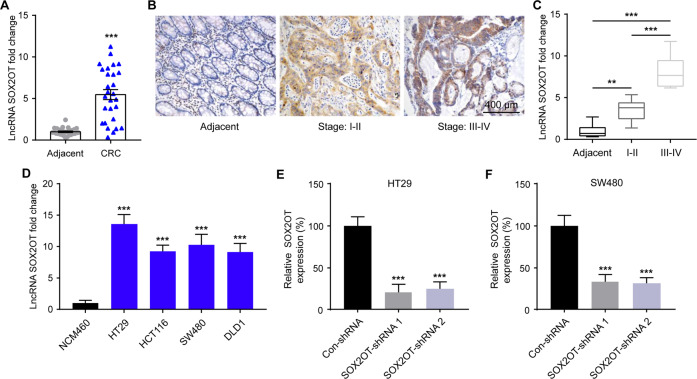
LncRNA SOX2OT expression was upregulated in CRC. **A** qRT-PCR showing the expression level of SOX2OT in CRC tissues and adjacent non-tumor tissues, ***P < 0.001. **B** Representative IHC images of staining of SOX2OT in different TNM stages in tumor and adjacent non-tumor tissues respectively, scale bar = 400 μm. **C** qRT-PCR showing the expression level of SOX2OT in different TNM stages, **P < 0.01, ***P < 0.001 vs. adjacent. **D** qRT-PCR showing the expression level of SOX2OT in CRC cell lines, ***P < 0.001 vs. NCM460. **E**, **F** shRNAs were used to knock down SOX2OT in HT29 and SW480 cells, ***P < 0.001 vs. Con-shRNA. Data were expressed as mean ± SD. All experiments were repeated three times.

To investigate the expression of SOX2OT in human CRC, we first analyzed SOX2OT in 28 paired colorectal tumor tissues. SOX2OT expression levels were indicative of a much higher expression in CRC tissue than in non-tumor adjacent tissues (Fig. 1A). SOX2OT was visualized with an ISH probe and it was found then the expression of SOX2OT was highly expressed in low-grade and high-grade CRC tissues even displayed stage-dependent higher expression manner (Fig. 1B) and the qRT-PCR results further confirmed these results (Fig. 1C). In addition, we measured the expression level of SOX2OT in CRC cell lines (HT29, HCT116, SW480, and DLD1) and normal colon mucosal epithelial cell line NCM460 by qRT-PCR. The results showed that SOX2OT expression was significantly increased in CRC cell lines cells compared with normal cell lines (Fig. 1D). These results showed that lncRNA SOX2OT is involved in CRC. To further investigate the function of SOX2OT in CRC, we selected HT29 and SW480 cells considering SOX2OT levels were the highest among the measured cell lines. We constructed shRNAs targeting SOX2OT and transfected two shRNAs into HT29 and SW480 cells to knock down SOX2OT expression. After transfection, shRNAs led to decreased expression of SOX2OT expression in HT29 and SW480 cells and these two shRNAs displayed a similar efficiency (Fig. 1E, F).

### SOX2OT knockdown inhibits CRC cell tumorbiology behavior in vitro

**Fig. 2:**
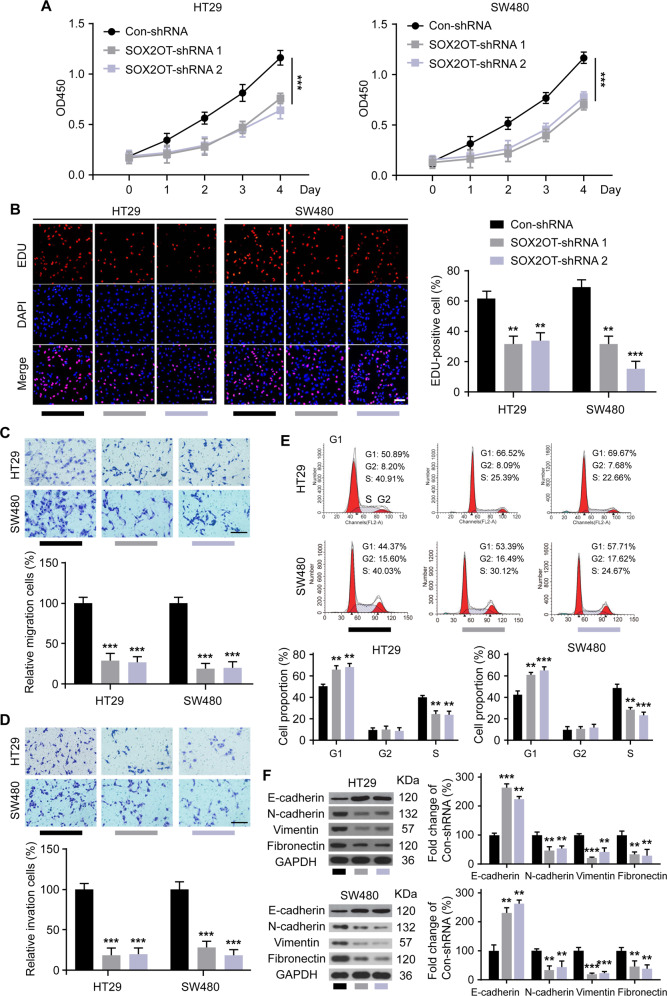
Knockdown of lncRNA SOX2OT inhibits the tumorigenicity of CRC cells in vitro. **A** CCK-8 assay was performed to determine the cell viability of HT29 and SW480 cells transfected with SOX2OT-shRNAs or negative control (Con-shRNA). **B** EDU assay was performed to measure the cell proliferation of HT29 and SW480 cells transfected with SOX2OT-shRNAs or negative control (Con-shRNA), scale bar = 50 μm; Transwell assays were conducted to showed cell migration and invasion in HT29 and SW480. **C**, **D** Cells transfected with SOX2OT-shRNAs or negative control (Con-shRNA), scale bar = 50 μm. **E** Cell cycles were analyzed with Flow cytometry in transfected HT29 and SW480 cells. **F** Transfected HT29 and SW480 cells were respectively applied to western blot to detect the expression levels of E-cadherin, N-cadherin,vimentin, and fibronectin. ***P < 0.001 vs. Con-shRNA. Data were expressed as mean ± SD. All experiments were repeated three times.

Cell viability and proliferation were evaluated by CCK8 assay and EDU assay, respectively. And, the results revealed that silencing of SOX2OT significantly suppressed cell viability and proliferation of HT29 and SW480 cells (Fig. 2A, B). Transwell chamber assays were conducted to further confirmed the effect of SOX2OT on CRC cell migratory capacity and invasive ability. SOX2OT knockdown decreased cell migration and invasion in both HT29 and SW480 cell lines (Fig. 2C, D), which was consitantly in line with the expectations. Given the importance of quiescence/senescence in CRC progression, to better understand the generated phenotype upon SOX2OT silencing, we performed a cell cycle analysis. The results showed the though minimal difference of G2 phase was found between Con-shRNA and SOX2OT-shRNAs in HT29 or SW480 cells (Fig. 2E), the percent of SOX2OT-shRNAs cells in the S phase was lower compared with that of Con-shRNA. In addition, we observed that there was a higher percentage of cells expressing SOX2OT-shRNAs in the G1 phase compared with Con-shRNA, indicating that SOX2OT silencing results in cell cycle arrest of CRC cells. We also analyzed the effect of SOX2OT knockdown on apoptosis of CRC cells. We found that SOX2OT silencing significantly induced the apoptosis of CRC cells (data not shown). Tumor metastasis is a tightly correlated epithelial-mesenchymal transition (EMT), it would be interesting to see the characterization of EMT markers upon SOX2OT silencing. So we examined the expression of EMT markers, including E-cadherin, N-cadherin, vimentin, and fibronectin. The WB results demonstrated that SOX2OT silencing markedly changed the protein expression levels of the above EMT markers (Fig. 2F). Taken together, these results indicated that knockdown SOX2OT decreased the tumorigenicity of CRC cells in vitro at least partly via modulating EMT.

### SOX2OT knockdown inhibits CRC growth in vivo

**Fig. 3:**
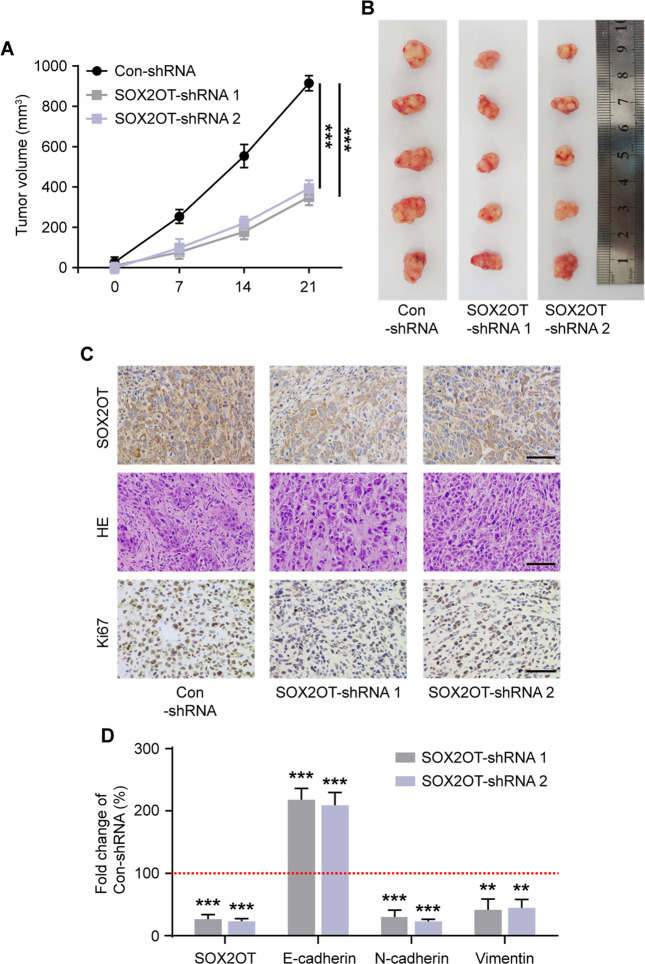
Effect of SOX2OT knockdown on CRC growth in vivo. **A** The transplanted tumor volume growth curves of nude mice were determined at different time points. **B** Images of subcutaneous transplanted tumors in nude mice. **C** Representative ISH staining of SOX2OT, HE staining, and IHC staining of Ki67 from the transplanted tumor. **D** Relative mRNA expression of SOX2OT, E-cadherin, N-cadherin, and vimentin in transplanted tumor. Scale bar = 50 μm. ***P < 0.001 vs. Con-shRNA. Data were expressed as mean ± SEM. All experiments were repeated three times.

To further probe the effect of SOX2OT in vivo, we subcutaneously injected SOX2OT-shRNAs and Con-shRNA stably transfected CRC cells into the back of the nude mice and observed the volume of tumors formed subcutaneously. Following the *3*R principles in animal use, we only injected HT29 cells. SOX2OT knockdown repressed tumor formation obviously compared with control cells (Fig. 3A, B). The ISH staining results confirmed that SOX2OT expression were decreased in SOX2OT-shRNAs tumors tissues (Fig. 3C). Under the light microscope, HE staining results showed that the cells of the SOX2OT knockdown group had the characteristics of more regular cell shape and degree of differentiation compared with the Con-shRNA group (Fig. 3C), immunohistochemical staining showed that expression of the proliferation marker Ki-67 was lower in SOX2OT knockdown tumors than control tumors (Fig. 3C). We further examined the correlation between the expression of SOX2OT and the EMT phenotype in vivo. RT-qPCR results showed SOX2OT silencing markedly changed the expression levels of E-cadherin, N-cadherin, and vimentin in the tumor tissues of nude mice (Fig. 3D). Collectively, these results confirm the oncogenic activity of SOX2OT in CRC in vivo.

### miR-194-5p is a target of SOX2OT and exhibited low expression in CRC

**Fig. 4:**
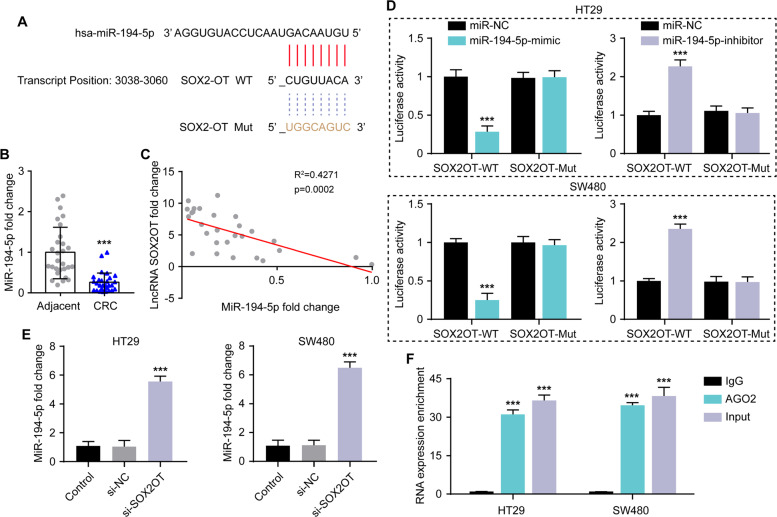
SOX2OT targeted miR-194-5p directly in CRC. **A** Putative binding site between SOX2OT and miR-194-5p through starBase v2.0. online software. **B** qRT-PCR was performed to determine miR-194-5p expression in 28 pairs of CRC tissues and adjacent non-tumor tissues, ***P < 0.001 vs. adjacent. **C** Pearson’s correlation analysis showed an inverse correlation between SOX2OT and miR-194-5p in CRC tissues. **D** Luciferase reporter assay for analysis of the interaction between SOX2OT and miR-194-5p in HT29 and SW480 cells, ***P < 0.001 vs. miR-NC. **E** Relative expression levels of miR-194-5p after silencing of SOX2OT in HT29 and SW480 cells by qRT-PCR, ***P < 0.001 vs. miR-NC. **F** SOX2OT enrichment in HT29 and SW480 cells is presented as fold enrichment in Ago2 relative to IgG immunoprecipitates, ***P < 0.001 vs. miR-NC. Data were expressed as mean ± SD. All experiments were repeated three times.

To investigate the target of SOX2OT, we applied the online software starBase v2.0. to predict the potential miRNAs that interacted with SOX2OT. The bioinformatics analysis revealed that miR-194-5p has putative binding sites with SOX2OT, thus it might be a potential target of SOX2OT (Fig. 4A). Furthermore, miR-194-5p was downregulated in CRC (Fig. 4B) and negatively correlated with SOX2OT level in CRC tissues (Fig. 4C). Dual-luciferase reporter assay showed a decrease in luciferase activity in CRC cells cotransfected with miR-194-5p-mimics and SOX2OT 3′-UTR-WT while there was an increase in luciferase activity in CRC cells cotransfected with miR-194-5p-inhibitor and SOX2OT 3′-UTR-WT compared with that in HT29 cells cotransfected with miR-NC and SOX2OT 3′-UTR-WT (Fig. 4D), suggesting that miR-194-5p was a direct bind site of SOX2OT in CRC. In a further qRT-PCR experiment, we also noticed that knockdown of SOX2OT increased the miR-194-5p expression in HT29 and SW480 cells (Fig. 4E). Furthermore, RNA-binding protein immunoprecipitation experiments results showed that SOX2OT were significantly enriched in immunoprecipitates of Ago2 isolated from HT29 and SW480 cells (Fig. 4F).

### SOX2OT affected CRC cell proliferation, migration, and invasion by regulating miR-194-5p

**Fig. 5:**
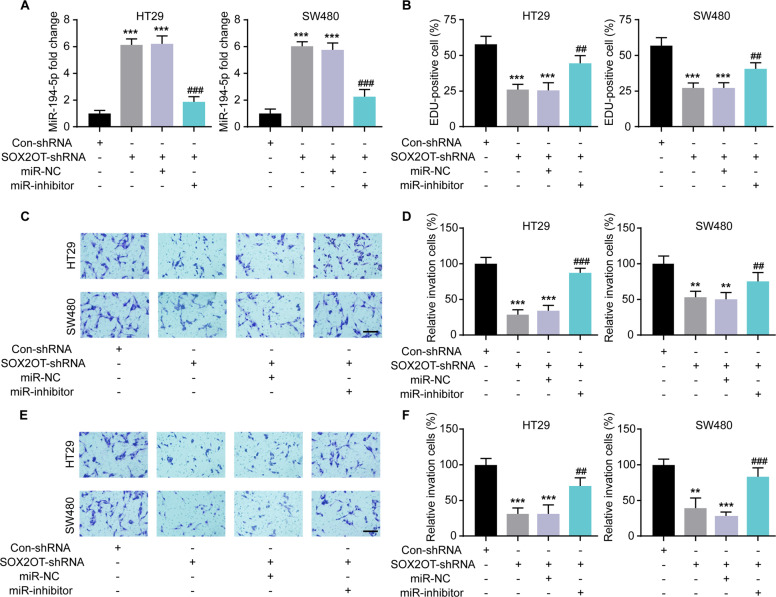
Down-regulation of miR-194-5p counteracted the tumor suppressive effects of sh-SOX2OT in CRC cells. **A** qRT-PCR was used to detect the expression of miR-194-5p in CRC cells after transfecting with SOX2OT-shRNA and/or miR-194-5p-inhibitor (miR-inhibitor); functional rescue experiments were carried out in HT29 and SW480 cells including EDU assay (**B**), transwell migration assay (**C**), (**D**), transwell invasion assay (**E**), (**F**). **P < 0.01, ***P < 0.001 vs. Con-shRNA; ##*P* < 0.01, ###*P* < 0.001 vs. SOX2OT-shRNA+miR-NC. Scale bar = 50 μm. Data were expressed as mean ± SD. All experiments were repeated three times.

The above results suggested that SOX2OT may affected CRC cells by downregulating miR-194-5p. Whether SOX2OT affected CRC cells via regulating miR-194-5p was investigated next. As the previous result showed, down-regulation of SOX2OT substantially increased miR-194-5p expression in CRC cells, thus SOX2OT-silenced HT29 and SW480 cells were cotransfected with miR-194-5p-inhibitor (miR-inhibitor) or miR-NC, and changes in the proliferation, migration, and invasion were measured. Cotransfection with miR-194-5p-inhibitor restored the miR-194-5p levels in HT29 and SW480 cells that were increased by SOX2OT knockdown (Fig. 5A). Subsequently, the results of functional experiments demonstrated that the effect of SOX2OT knockdown on HT29 and SW480 cell proliferation (Fig. 5B), migration (Fig. 5C), and invasion (Fig. 5D) were almost abolished after the downregulation of the miR-194-5p.

### SOX5 is targeted by miR-194-5p and SOX5/ miR-194-5p axis regulates tumor behaviors of CRC cells

**Fig. 6:**
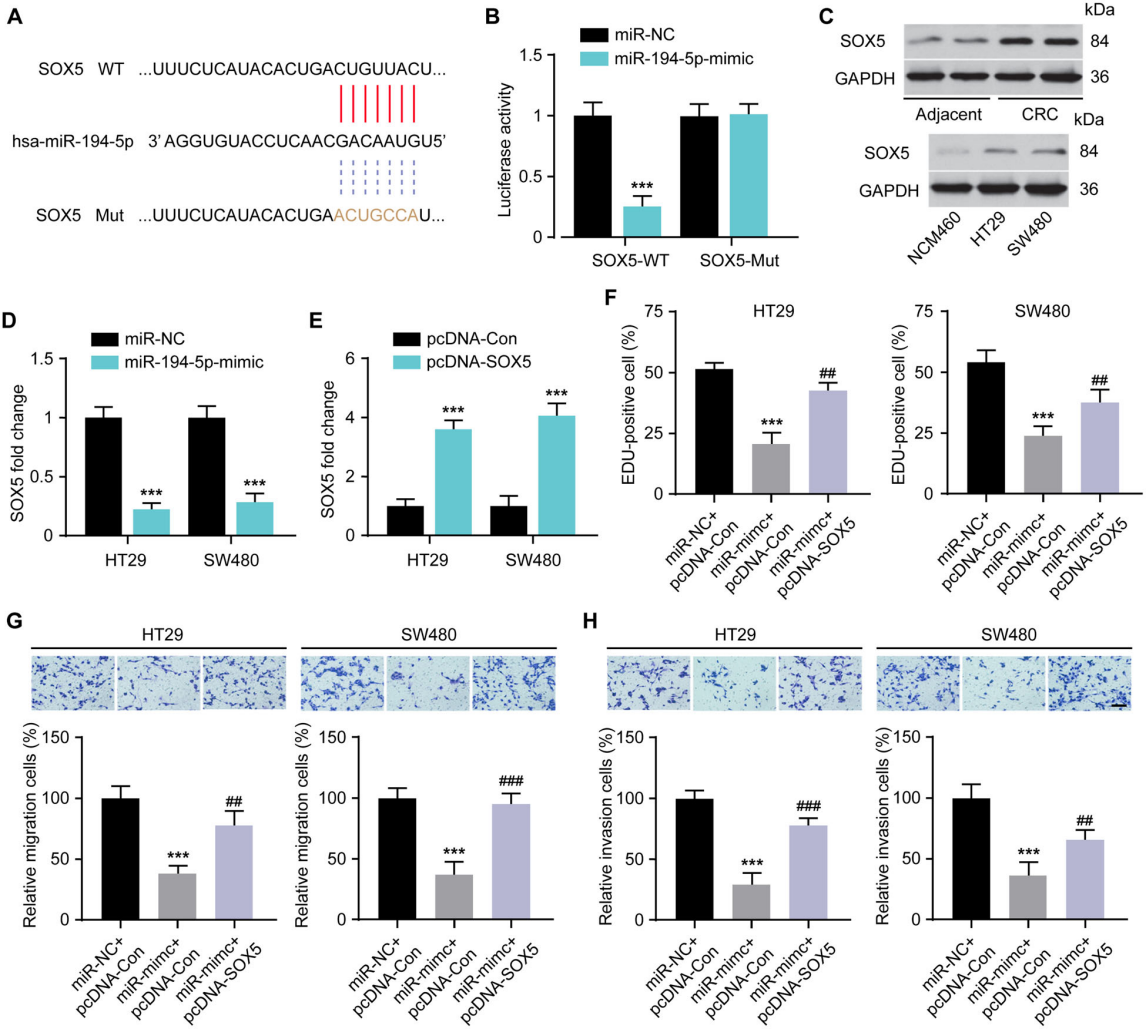
SOX5 is a direct target of miR-194-5p and its upregulation reverses the impacts of miR-194-5p on CRC cells. **A** The putative binding site between SOX5 and miR-194-5p through prediction of TargetScan online software. **B** luciferase reporter assay for analysis of the interaction between SOX5 and miR-194-5p, ***P < 0.001 vs. miR-NC. **C** western blotting was performed to detect the protein expression levels of SOX5 in CRC tissues and CRC cell lines. **D** qRT-PCR was performed to determine the effect of miR-194-5p-mimic in decreasing SOX5 expression in HT29 and SW480 cells, ***P < 0.001 vs. miR-NC. **E** HT29 and SW480 cells were transfected with pcDNA3.1-SOX5 and the fold changes in the expression of SOX5 was analyzed, ***P < 0.001 vs. pcDNA-Con; EDU (**F**), migration (**G**), and invasion (**H**) assays were conducted respectively in HT29 and SW480 cells mentioned above, ***P < 0.001 vs. miR-NC + pcDNA-Con; ^##^*P* < 0.01, ^###^*P* < 0.001 vs. miR-mimic + pcDNA-Con. Scale bar = 50 μm. Data were expressed as mean ± SD. All experiments were repeated three times.

Next, we predicted potential target genes of miR-194-5p by bioinformatics prediction (TargetScan and Starbase) method and identified that SOX5 3′-UTR contains a potential binding site with miR-194-5p thus selected for further analyses (Fig. 6A). After construction of luciferase plasmids SOX5 3′-UTR-WT and corresponding SOX5 3′-UTR-Mut, they were cotransfected with miR-194-5p-mimics or miR-NC in HT29 cells. The results showed that miR-194-5p-mimic significantly repressed the luciferase activity in HT29 cells transduced with SOX5 3′-UTR-WT reporter (Fig. 6B), which indicated that SOX5 was a potential target gene of miR-194-5p. Subsequently, the expression of SOX5 in CRC tissue and CRC cell line was determined by WB. The protein levels of SOX5 were remarkably elevated in CRC tumor tissue and CRC cells (Fig. 6C). To confirmed whether miR-194-5p regulated SOX5 expression, we detected expression levels of SOX5 in HT29 and SW480 cells after transfection of miR-194-5p-mimics, qRT-PCR and analysis revealed that miR-194-5p overexpression significantly inhibited the expression of SOX5 in CRC cells (Fig. 6D). We next explored whether SOX5 contributes to the miR-194-5p mediated proliferative, migratory, and invasive potentials of CRC cells.

The transfection of pcDNA3.1-SOX5 was verified by qRT-PCR and the results showed that it markedly increased the level of SOX5 in HT29 and SW480 cells (Fig. 6E). Functionally, the ectopic expression of SOX5 in HT29 and SW480 cells abrogated the tumor inhibitory behaviors induced by miR-194-5p unregulated. Cell proliferation (Fig. 6F), migration (Fig. 6G), and invasion assay (Fig. 6H) results revealed that SOX5 restoration rescued the inhibitory influences of miR-194-5p-mimics on HT29 and SW480 cells. On the basis of the above results, SOX2OT/miR-194-5p/SOX5 axis showed great effects on regulating oncogenic behaviors of CRC cells.

## Discussion

Among the recent CRC reseach works, the vital role of lncRNA in tumorigenesis and progression has been recognized by the scientific community with the help of clinical bioinformatics analysis combined with functional research. Improved understanding the role of lncRNA in tumor biology has led to advances in the diagnosis, classification, prognostication and novel treatment of patients with CRC. In the present study, we aimed to explore the role SOX2OT in CRC development, which has shown to be significantly increased both in CRC tissues and cell lines. Functional assays confirmed that SOX2OT was an oncogene in CRC development and may be utilized as a prognostic indicator for CRC. SOX2OT increased the level of SOX5 and induced malignant biological behaviors in CRC by inhibiting miR-194-5p expression. Thus, based on our results, the following conclusions can be drawn: SOX2OT functions as an oncogene in CRC and should be considered a potential prognostic indicator.

In 2009, Amaral et al. found that SOX2OT is a key regulator of pluripotency embedded stable transcript expressed during vertebrate embryogenesis developmen^10^. After embryonic development, SOX2OT is abnormally expressed in many diseases and plays an important role in the regulation of malignant biological behavior of many tumors and is closely related to the prognosis of tumor patients^19^. Over-expressed SOX2OT has also been confirmed in patients with small-cell lung cancer^20^, hepatocellular carcinoma^21^, breast cancer, etc. Functionally, consistanted with our results, SOX2OT performs oncogenic actions in bladder cancer^22^, pancreatic cancer^23^, gastric cancer^24^ cells. Similar to a previous study which showed that SOX2OT’s qRT-PCR results in CRC^12^, we further confirmed the upregulation of SOX2OT in CRC, and higher expression of SOX2OT was correlated with advanced TNM stage. In this study, silencing lncRNA SOX2OT influenced various tumor cellular behavioral processes. The molecular mechanism of SOX2OT in tumor progression is quite intricate. MiRNA-mediated targeting strategy of lncRNAs is well documented in the mechanistic control of gene expression. SOX2OT had shown to promotes hepatocellular carcinoma development through miR-122-5p/PKM2 axis^21^. In non-small-cell lung cancer, SOX2OT had shown to interact with miR-132 to promotes cancer metablism^25^. It is possible to speculated that the up-regulated SOX2OT in CRC share similar regulatory mechanisms, there may exist miRNA-mediated mechanism that miRNA as a sponge to attenuate SOX2OT-mediated activity.

Bioinformatics analysis recognition binding site sequences on SOX2OT revealed the presence of thousands miRNAs binding sites. Among them, miR-194-5p stood out through the detailed relevant studies analysis using NCBI hand searching. Our results confirmed that miR-194-5p was significantly downregulated in the CRC tissues compared with the normal tissues, and its expression was negatively correlated with SOX2OT, indicating that miR-194-5p is a direct target of SOX2OT in CRC. Luciferase assays validate its direct binding and full-length transcription regulating ability with SOX2OT. In addition, we confirmed whether miR-194-5p mediated the counteractive impacts of SOX2OT knockdown in CRC cell growth and metastasis. Our data revealed that, despite the fact that knockdown of SOX2OT suppressed CRC cell proliferation, migration, and invasion, administration of miR-194-5p-inhibitor could reverse the impact induced by the SOX2OT knockdown. In this way, SOX2OT promoted CRC progression through the inhibition of miR-194-5p. This SOX2OT/miR-194-5p feedback loop had also been confirmed in other cancer^26,27^ which further support our conclusions.

As shown in previous studies, miR-194-5p acted as a tumor suppressor in many cancers, such as ovarian cancer^28^, bladder cancer^29^, glioblastoma multiforme^30^. Consistent with the aforementioned literature, miR-194-5p exerts an inhibitory effect in CRC. It was well recognized that miRNAs could directly influence the transcription of the target mRNAs. We used several different miRNAs target predicting software and selected out SOX5 as a downstream target of miR-194-5p, cause SOX5 belongs to the same family of SOX, which mediating the cell proliferation by binding HMG domine, which is vital for tumorgenesis^31^, and SOX2OT had shown its regulation on the stemness phenotype of cervical cancer cells and bladder cancer by modulating SOX2^22,32^. We used luciferase reporting assay to validate SOX5 as a novel target gene of miR-194-5p. SOX5 were reported to be hypermethylated in CRC and antineoplastic drugs could restored its expression in CRC cells^33^. However, the effect of lncRNA on its expression has not been reported previously. For the first time, this study found that SOX2OT may be an important regulator of SOX5 in CRC, SOX2OT can reduce the inhibitory effect of miR-194-5p on SOX5 through sponge adsorption of miR-194-5p, thus promoting the proliferation, migration and invasion of CRC cells, further research with the aim of exploring the underlying mechanisms is recommended.

Taken together, we reported for the first time that SOX2OT/miR-194-5p/SOX5 pathway is a therapeutic target for CRC. Our in vivo animal xenograft experiments results further showed that SOX2OT interference inhibited the growth of tumor xenografts and regulating the expression of Ki-67. Novel molecular intervention strategies therapies in CRC treatment should not only inhibit of growth but also metastasis, thereby contribute to reduce malignancy and improved survival. Through our study, we have preliminarily demonstrated that SOX2OT, working as an oncogene, promoted proliferation, migration and invasion by upregulating SOX5 via acting as a ceRNA of miR-194-5p in CRC. We propose that SOX2OT and miR-194-5p may serve as potential biomarkers for the diagnosis of CRC.

## Data Availability

The datasets used or/and analyzed during the current study are available from the corresponding author on reasonable request.
